# Immune–reproductive cross talk in mosquitoes: molecular pathways and energy homeostasis

**DOI:** 10.1186/s13071-026-07555-2

**Published:** 2026-07-03

**Authors:** Feifei Zheng, Caizhi Zhao, Xin Li, Tingting Liu, Shun Wang, Zhilong Liu, Shasha Yu, Ying Wang

**Affiliations:** 1 Department of Tropical Medicine, College of Military Preventive Medicine, Army Medical University, No. 30 Gaotanyan Street, Shapingba District, Chongqing, 400038 China; 2 School of Public Health, The Key Laboratory of Environmental Pollution Monitoring and Disease Control, Ministry of Education, Guizhou Medical University, Guiyang, 561113 China; 3 Department of Pathogenic Biology, Army Medical University, Chongqing, China

**Keywords:** Reproduction, Immunity, Juvenile hormone, 20-hydroxyecdysone, Autophagy, Metal ions

## Abstract

In the field of global health, mosquito-borne infectious diseases (such as malaria, dengue fever, and Zika) remain serious public health concerns. Recent research has emphasized that mosquitoes must coordinate their reproductive output and immune defenses under limited energy and nutritional conditions, forming a tightly regulated trade-off. This process involves complex signaling pathways and stage-specific molecular mechanisms. Key factors, such as juvenile hormone (JH), 20-hydroxyecdysone (20E), autophagy pathways, immune-regulatory genes, and metal ions, can potentially influence this equilibrium state, thereby either promoting or inhibiting the occurrence and development of mosquito-borne infectious diseases. However, the exact mechanism underlying this regulation remains unclear. This review summarizes recent findings on how these components interact at the molecular and physiological levels, with the aim of developing a rational framework for identifying potential molecular targets and developing new strategies for vector control.

**Graphical Abstract:**

**Figure:**
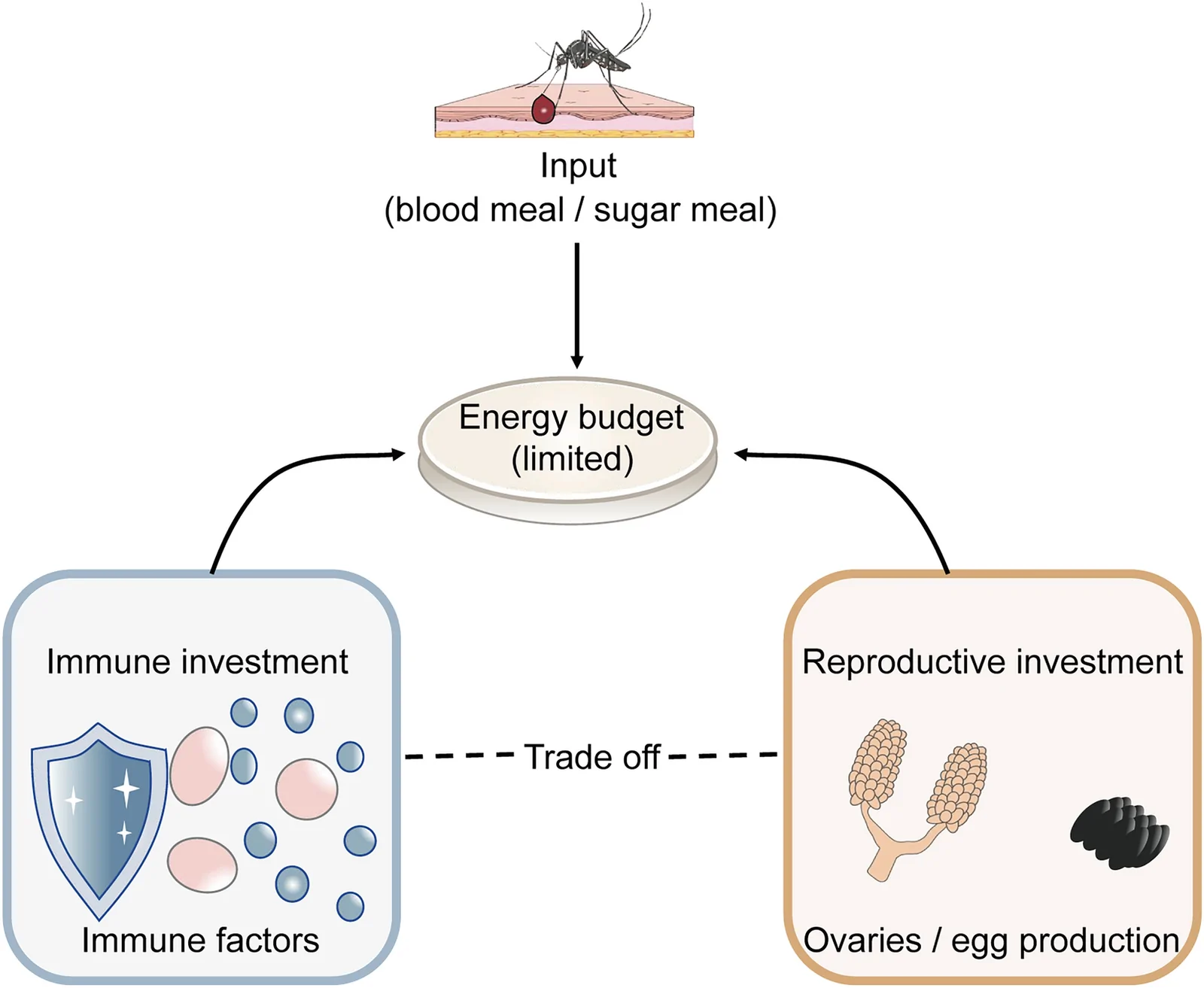

## Background

Malaria parasites, dengue virus (DENV), Zika virus (ZIKV), and chikungunya virus (CHIKV), as major arthropod-borne pathogens responsible for several serious infectious diseases, present substantial and enduring threats to global health, particularly in subtropical and tropical regions [1–3]. Mosquito population density and vectorial transmission capacity are key factors determining the spread and prevalence of these diseases. The reproductive capacity and innate immune system determine the efficiency of population maintenance and pathogen transmission, respectively.

Recent advances in molecular biology have afforded us a comprehensive understanding of mosquito immune defense and reproductive processes, encompassing the characterization of key signaling pathways and regulatory molecules governing these biological events. The mosquito innate immune system is highly complex, with diverse innate defense mechanisms acting primarily at critical tissues (midgut, hemocoel, salivary glands, and fat body) to fend off pathogen invasion [4]. Humoral immune responses depend on the regulation of various effector molecules, including antimicrobial peptides (AMPs) and complement-like proteins (thioester-containing proteins, TEPs). In contrast, the cellular immune response is mediated through phagocytosis and the encapsulation of blood cells to eliminate pathogens [5–8]. These immune signaling pathways, including Janus Kinase-Signal Transducer and Activator of Transcription (JAK-STAT), Immune deficiency pathway (Imd), Toll pathway (Toll), and RNA interference (RNAi), serve as core mediators in defending mosquitoes against a broad range of pathogens, including bacteria, fungi, viruses, and parasites [9, 10]. RNAi pathways effectively identify and silence viral gene expression through small interfering RNA (siRNAs), microRNAs (miRNAs), and piwi-interacting RNA (piRNAs), thereby limiting viral replication and transmission [9]. By contrast, the Toll, JAK-STAT, and Imd pathways govern the expression of immune-related genes by activating signaling cascades upon recognition of pathogen-associated molecular patterns (PAMPs) [4, 10]. The reproductive processes in mosquitoes are governed by multiple regulatory layers, ranging from environmental cues to molecular mechanisms. At the organismal level, environmental conditions such as temperature and light–dark cycles directly influence reproductive behavior and subsequently impact disease transmission [11, 12]. At the molecular level, mosquito reproduction is regulated by hormones, signaling proteins, and gene regulatory networks. For instance, juvenile hormone (JH) and 20-hydroxyecdysone (20E) serve as the primary endocrine regulators that sequentially govern previtellogenic and vitellogenic development, respectively, while leucine aminopeptidase 1 is essential for egg laying and hatching success [13]. In addition, miRNAs play a role in controlling signaling pathways linked to mosquito reproduction [14, 15].

Despite substantial advances in elucidating mosquito immunity and reproductive physiology independently, the mechanistic cross talk between these two essential biological processes remains incompletely defined, making this a complex yet pivotal research area in vector biology. This review centers on the molecular and physiological intersection of these two regulatory systems, and current evidence indicates that the interplay between JH, 20E, insulin/insulin-like growth factor signaling (IIS), and target of rapamycin (TOR) pathway orchestrates the trade-off between mosquito reproduction and immunity [16–20]. Precise modulation of these signaling cascades can alter the dynamic balance between reproductive output and immune defense [21–24], while autophagy also serves as a key regulatory node in coordinating these interconnected biological processes [25–27]. Furthermore, metal ions including iron, zinc, and copper exert dual regulatory functions in mosquito immunity and reproduction [28–32]. Thus, elucidating this integrated, multi-layered regulatory network—including hormonal signaling, nutrient-sensing pathways, autophagy, and metal ion modulation—not only deepens our understanding of mosquito biological traits but also provides a solid theoretical basis for developing more effective control strategies for mosquito-borne infectious diseases.

## Immune–reproduction interaction

### Energy homeostasis

Mosquito nutritional physiology serves as a fundamental upstream regulator, coordinating systemic energy homeostasis and governing the trade-off between immune defense and reproductive physiology. Both reproductive and immune processes are energetically costly, and the two often compete for limited endogenous nutritional resources [33]. Studies in *Drosophila melanogaster* have confirmed that immune activation reduces reproductive output, while mating and oviposition suppress immune gene expression and compromise pathogen resistance [34, 35]. This evolutionary trade-off is highly conserved in mosquitoes. Infection of *Aedes aegypti* with *Strigomonas culicis* disrupts midgut redox homeostasis and drastically reduces egg production [36]. Similarly, immune stimulation and malaria parasite infection impair the reproductive capacity of *Anopheles gambiae* by triggering follicle apoptosis [37, 38], indicating that immune activation diverts energetic resources away from reproduction. Constitutive overexpression of activated AMP-activated protein kinase (AMPK) in *Anopheles stephensi* reduces egg production while enhancing *Plasmodium* resistance, providing direct evidence that energy allocation imbalance shapes mosquito immune and reproductive phenotypes [39].

Nutritional status is a central determinant of immune-reproductive trade-offs in mosquitoes, governing both resource availability and immune activation thresholds [40, 41]. In *Ae. aegypti*, dietary restriction downregulates key immune pathways (Toll, JAK-STAT, Imd) and antimicrobial peptide expression, weakening innate immune defenses. Nutrient scarcity not only reduces female egg production [40], but also correlates with impaired immune function [34, 40], suggesting that nutrient availability directly balances reproductive investment and immune defense. TOR signaling is reduced under nutrient deprivation/starvation [42, 43]. Starvation additionally represses juvenile hormone acid methyltransferase (JHAMT) expression, thus suppressing JH biosynthesis. Concurrently, increased triglycerides mobilization and β-oxidation redirect energy resources away from reproductive processes, resulting in lipid depletion and increased oocyte resorption rates [41]. Beyond TOR and JH signaling, 20E-induced miR-276 reshapes amino acid catabolism by targeting branched-chain amino acid aminotransferase (BCAT), thereby fine-tuning post-blood-meal metabolic allocation and influencing both reproductive cycling and *Plasmodium falciparum* development [44]. Interestingly, *P. falciparum* has evolved adaptive strategies to optimize its replication by scavenging host-derived lipids, with no significant impact on mosquito egg development. However, experimental interference with ovulation has been shown to accelerate parasite development and enhance infectivity [45–47]. This indicates that the parasite may affect how the host distributes its resources. These findings emphasize that energy homeostasis, influenced by diet, hormonal signals, and parasite manipulation, constitutes the metabolic basis of mosquito immune–reproductive interactions.

In addition, nutritional inputs shape gut microbial dynamics, linking metabolic status to immune regulation through microbiota-dependent mechanisms. In *An. stephensi*, blood feeding enriches the gut microbial community and enhances anti-*Plasmodium* immune responses, whereas *Plasmodium vivax* infection reshapes microbiota composition and disrupts immune homeostasis, supporting a tripartite interaction between nutrition, microbiota, and parasite survival [48]. Parasite infection also disturbs host metabolic allocation. Modulation of sterol carrier protein during *P. vivax* infection suggests altered sterol transport and resource distribution, linking parasite development with reproductive physiology [49]. These findings suggest that nutritional inputs coordinate microbiota dynamics, immune activity, and metabolic allocation, thereby influencing both reproductive output and pathogen development.

Beyond cell-intrinsic trade-offs, mosquitoes orchestrate systemic energy allocation across tissues in a nutrition-dependent manner through inter-organ communication, predominantly via the fat body–ovary and gut–ovary axes. Along the fat body–ovary axis, blood meal–elicited metabolic and endocrine signals drive vitellogenin synthesis in the fat body and support subsequent oocyte maturation [19, 42, 50, 51]. Recent time-resolved phosphoproteomic profiling of the mosquito fat body revealed coordinated activation of nutrient-sensing, ribosome biogenesis, and autophagy pathways after blood feeding, highlighting the fat body as a dynamic signaling nexus integrating metabolic and developmental cues [52]. More recently, single-cell transcriptomic analyses have uncovered cellular heterogeneity within the mosquito fat body and its dynamic response to blood ingestion, including a previously uncharacterized fat body-yolk cell population linked to reproductive programs, providing a cellular basis for coordinated fat body–ovary regulation [53]. At the molecular level, fat body–enriched factors such as circRNA-407 further support the role for the fat body in regulating ovarian development [54]. Along the gut–ovary axis in *Ae. aegypti*, JH contributes to the establishment of a midgut microbial community that enhances female fecundity, in part by regulating midgut immune homeostasis [55]. Similar principles of inter-organ metabolic regulation have been described in *Drosophila*, where ovary-derived steroid signaling remodels intestinal physiology to promote reproductive output [56].

### Endocrine and signaling regulatory networks governing reproduction and immunity

#### Hormonal cross talk: JH and 20E

**Fig. 1:**
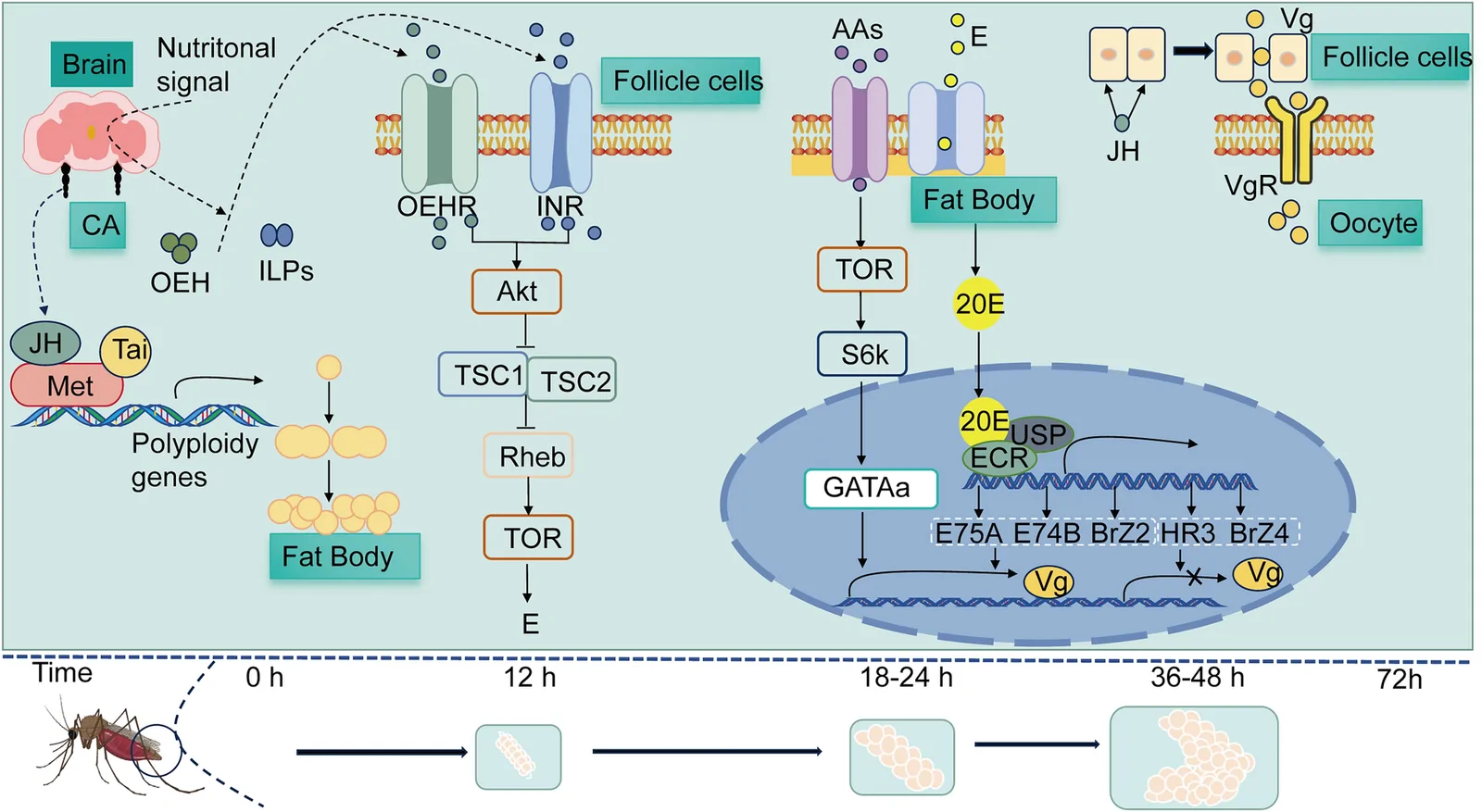
Hormonal and nutritional regulation of vitellogenesis in mosquitoes. During the pre-blood meal stage, JH secreted by the CA acts on the fat body through the Met/Tai complex, rendering it competent for subsequent vitellogenin synthesis. Following a blood meal, OEH and ILPs released from the brain activate ovarian E synthesis. Between 12–24 h post-blood meal, E is converted into 20E in the fat body, which binds to EcR/USP to induce early gene expression (*E75A, E74B, BrZ2*), followed by late genes (*HR3, BrZ4*), which terminate vitellogenesis and initiate autophagy. Amino acids activate TOR/S6K signaling to promote Vg expression via GATAa. Vg is taken up by oocytes via VgR-mediated endocytosis assisted by JH-regulated follicular patency. Arrows indicate stimulatory effects; T-shaped lines represent inhibition

In mosquitoes, JH and 20E orchestrate the reproductive cycle in a time-dependent manner through a cascade of endocrine and metabolic signals. This dynamic hormonal interplay governs fat body polyploidization, vitellogenin (Vg) synthesis, and oocyte maturation within a tightly regulated timeframe (Fig. 1). During the pre-blood meal stage (PE), JH secreted by the corpora allata (CA) binds to its receptor, methoprene-tolerant (Met). The resulting JH-Met complex subsequently dimerizes with its co-activator Taiman (Tai), forming the transcriptionally active JH-Met/Tai complex [57]. This complex initiates the transcription of genes associated with fat body polyploidization, thereby rendering the fat body competent to respond to subsequent vitellogenic stimulation and enhancing its biosynthetic capacity for vitellogenesis [58, 59]. Within the first 12 h after a blood meal, the mosquito brain secretes neuropeptides, such as ovarian ecdysteroidogenic hormone (OEH) and insulin-like peptides (ILPs), which stimulate the ovaries to synthesize and release ecdysone (E) [33, 60, 61]. From 12 to 24 h post-blood meal, the fat body converts E to its bioactive form 20E. High levels of 20E bind to the preformed heterodimer of the ecdysone receptor (EcR) and its co-receptor ultraspiracle (USP), inducing the expression of fat body early response genes (e.g., *E74B, E75A*, broad-complex zinc finger protein 2 [*BrZ2*]) [62, 63], which is subsequently followed by the transcription of late response genes including lipophorin (*Lp*) and vitellogenin (*Vg*). This process is supported by nutrient-sensing pathways, particularly amino acid-dependent TOR signaling [42, 50, 51, 64]. Amino acid availability promotes ILP signaling to support reproductive metabolism, while stage-specific regulators such as GATA transcription factors further fine-tune lipid allocation [18, 19, 65–67]. By ~ 36 h post-blood meal, as 20E levels decline to basal concentrations, the nuclear receptor EcR induces the expression of hormone receptor 3 (*HR3*) and broad-complex zinc finger protein 4 (*BrZ4*). In conjunction with JH, these transcription factors further suppress vitellogenesis, inhibit the TOR signaling pathway, and initiate fat body autophagy [63, 68]. This process drives functional remodeling of the fat body, priming the tissue for subsequent reproductive cycles. RNAi-mediated knockdown of *EcR* and autophagy-related genes (ATGs) results in the downregulation of the beta isoform of Fushi tarazu factor-1 (βFTZ-F1), which delays oocyte maturation and disrupts reproductive cycling [17, 63]. Lp mediates the transport of Vg from the fat body to the ovaries, a process essential for egg development [69]; in *An. gambiae*, reduced Lp expression alters Vg distribution and impairs oviposition efficiency [69]. JH additionally modulates ovarian function by activating G protein-coupled receptors (GPCRs) and Na⁺/K⁺-ATPase, which sustain follicular patency to facilitate Vg uptake [70]. Oocyte Vg uptake is mediated by Vg receptor (VgR)-dependent endocytosis, a process that promotes oocyte maturation [70]. Elevated JH levels protect ovarian cells by inhibiting caspase-mediated apoptosis, thereby mitigating ovarian atrophy [38]. Furthermore, JH regulates the expression of the ecdysone-induced protein 75 isoform RD (E75-RD) transcription factor in *Ae. aegypti*, which modulates miR-2940 activity and promotes the formation of healthy germ cells [71].

**Fig. 2:**
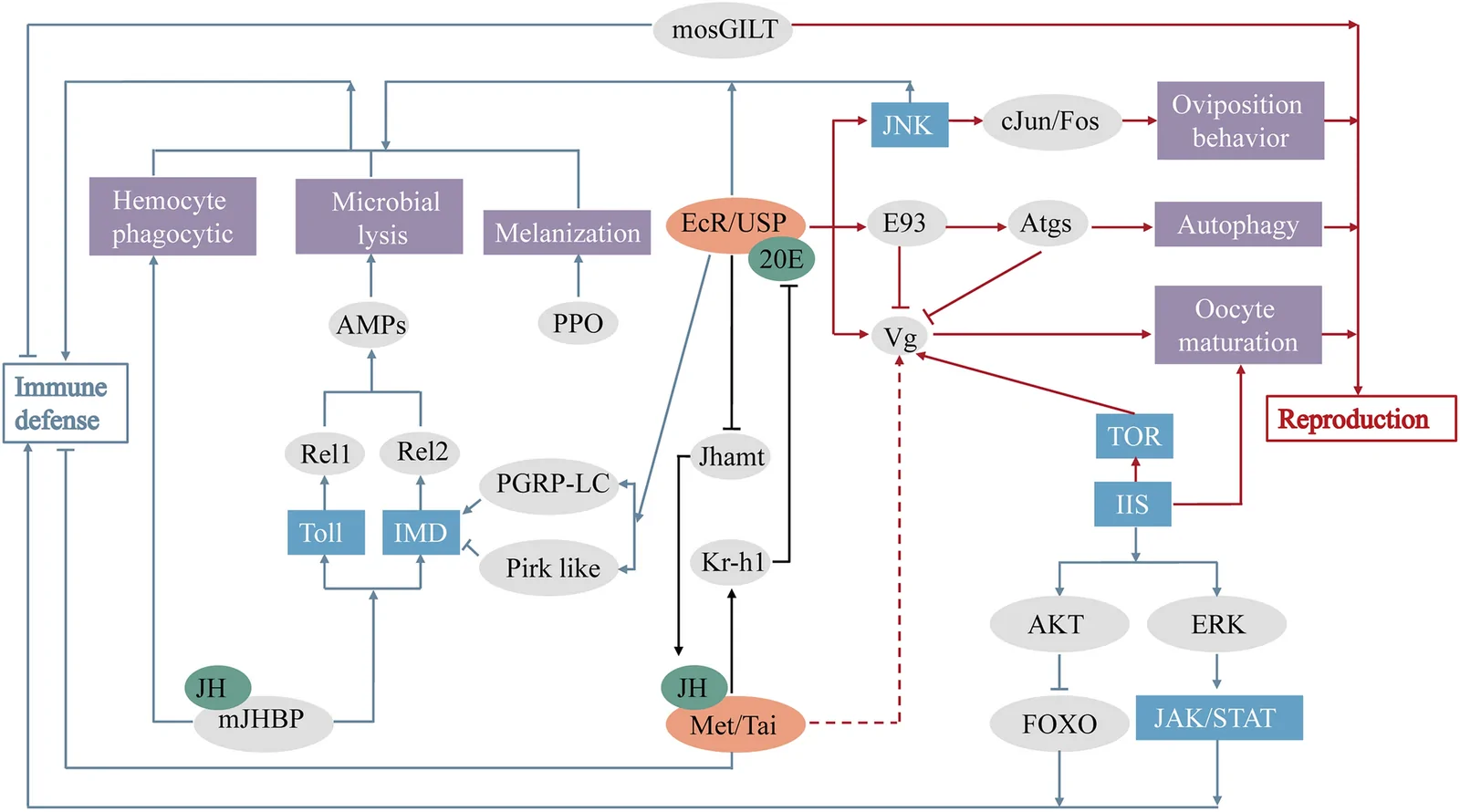
Hormonal and signaling pathways coordinating mosquito immune and reproductive functions. 20E binds the EcR/USP heterodimer and coordinately regulates mosquito immune and reproductive programs. 20E can enhance immune defense by promoting AMPs expression, melanization, and hemocyte phagocytic activity through multiple immune signaling pathways. In parallel, 20E contributes to reproductive transitions by inducing downstream factors such as E93 and ATGs, thereby supporting the switch from vitellogenesis to post-vitellogenic tissue remodeling. JH signals via the Met/Tai receptor complex and induces the JH-response factor Kr-h1; JH and 20E exhibit antagonistic cross talk, and JH/Kr-h1 may modulate EcR/USP-dependent transcription in a context-dependent manner. JH also shows phase-specific effects on immunity, generally suppressing basal immune activity, whereas the JH–mJHBP complex can stimulate immune responses by enhancing AMPs production and hemocyte functions. In addition, the IIS pathway promotes reproduction through TOR-mediated vitellogenesis and modulates immunity via FOXO and JAK-STAT signaling. MosGILT is included as an additional regulator linking immune defense and reproductive outputs. Arrow types and colors: → (Arrow): Activation; ⊣ (T-bar): Inhibition. Dashed lines: indirect regulation; red lines: reproductive pathways; blue lines: immune pathways; black lines: hormonal cross-regulation between 20E and JH; green ovals: Hormones; Orange ovals: hormone receptor complexes; light blue boxes: Signaling pathways; purple boxes: physiological processes; gray ovals: proteins and intermediate regulators

Beyond their canonical reproductive functions, JH and 20E orchestrate immune regulation through temporally distinct and mutually antagonistic signaling pathways (Fig. 2; Table 1). Krüppel homolog 1 (Kr-h1) mediates JH signaling to inhibit 20E biosynthesis, whereas 20E acts through its nuclear receptor complex EcR/USP to downregulate key genes involved in JH synthesis in CA [72, 73]. These reciprocal hormonal interactions extend to immune regulation, and JH generally exerts immunosuppressive effects under basal physiological conditions. Exogenous application of methoprene (a synthetic JH analog) can reduce AMP activation in infected *D. melanogaster* [72]. In addition, JH increases ZIKV load by modulating the expression of ribosomal proteins RpL23 and RpL27 [74], and attenuates the immune response by inhibiting phenoloxidase activity [75–77]. Interestingly, the complex formed by JH and mosquito juvenile hormone-binding protein (mJHBP) exhibits immunomodulatory activity: it not only stimulates hemocyte phagocytic activity but also upregulates AMP expression and activates Rel1/Rel2 via immune signaling cascades [8]. In contrast, 20E promotes immune defense. In *Drosophila* studies, 20E signals through EcR to regulate both peptidoglycan recognition protein LC (PGRP-LC)-dependent and -independent mechanisms, thereby inducing Imd signaling and AMP production [78]. In *An. gambiae,* 20E increases hemocyte phagocytic activity and upregulates leucine-rich repeat immune protein 9 (LRIM9) and prophenol oxidase (PPO), thereby enhancing resistance to *Plasmodium* [7, 21, 79]. However, during peak reproductive investment, 20E transiently suppresses immunity by inducing Pirk-like expression via the EcR/USP complex, thereby downregulating the Imd pathway [16]. Furthermore, inhibition of the TOR signaling pathway via rapamycin treatment or RNAi-mediated knockdown upregulates NF-κB transcription factor Rel2, which may counteract this immunosuppression [22]. Male-derived 20E also modulates immunity after mating. In *Anopheles*, high levels of 20E transferred during copulation promote ovarian development while reprogramming midgut immunity, increasing female susceptibility to *P. falciparum* [80]. Moreover, the male-specific *Anopheles* steroid 3-dehydro-20E (3D20E) becomes activated in mated females; this molecule not only suppresses female mating behavior but also induces genes that protect egg development under *Plasmodium* infection stress [81]. In addition, 20E-activated JNK signaling promotes oviposition behavior via its downstream effectors c-Jun/Fos. Knockdown of *JNK* impairs 20E-induced oviposition, whereas silencing of its negative regulator *puckered* enhances phosphorylated JNK (pJNK) levels and induced oviposition, even in unmated females [82].

**Table 1:** Representative immune–reproductive dual regulators in mosquitoes

Regulatory category	Pathway / factor	Key molecules	Dual functional role	References
Hormones	JH	Met/Tai, mJHBP, Kr-h1, RpL23/RpL27	Promotes lipid storage and vitellogenesis; suppresses immune responses (except mJHBP-JH complex which enhances immunity)	[33, 72–75]
	20E	EcR/USP, E74, E75, BrZ, Pirk-like, E93	Promotes germline stability, oocyte maturation and immune defense; transiently suppresses immunity during peak reproduction; initiates autophagy for reproductive transition	[7, 16, 26, 63, 79, 123–125]
Signaling pathways	IIS	ILPs, AKT, PTEN, GSK3	Regulates oocyte maturation, lipid metabolism, and antiviral immunity	[18, 23, 24, 43, 65, 83, 91, 92]
	TOR	S6K, GATAa, GATAr, FOXO	Responds to amino acids; regulates vitellogenin synthesis and immune gene expression	[42, 50, 51, 64, 65, 67]
	AMPK	AMPKα	Enhances resistance to *Plasmodium*; modulates reproductive output	[26, 39]
	JNK	puckered, pJNK	Required for 20E-induced oviposition; modulates immune signaling	[80–82]
Functional molecules	mosGILT	mosGILT, TEP1	Impairs ovarian development (via reduced 20E); its deletion enhances immune responses and modulates *Plasmodium* motility	[104–109]
	PGRP-SC2	PGRP-SC2	Maintains immune homeostasis (microbial recognition); its suppression in aged insects causes immune dysregulation and affects reproductive-related fitness	[93]
Metal ions	Iron	Ferritin, Transferrin	Essential for heme synthesis and reproduction; modulates *Plasmodium* infection and redox homeostasis	[110, 113, 119, 120]
	Zinc	Zn^2+^, Zn-dependent enzymes	Fluctuates during oocyte maturation; contributes to antiviral defense	[115, 117]
	Copper	Cu^2+^, PO	Supports melanization and eggshell/follicular maturation; chelation reduces *Plasmodium* transmission	[29, 31, 116, 118]
	Manganese	Mn^2+^	Induces cGAS-STING via oxidative stress; supports reproductive homeostasis	[110, 117, 122]

#### IIS balances reproduction and immune defense

The IIS pathway plays an important role in regulating mosquito immune responses and reproductive processes, thereby coordinating metabolic allocation between immune and reproductive processes (Fig. 2; Table 1). Insulin receptor activation initiates the PI3K/AKT signaling cascade via insulin receptor substrates (IRS), resulting in suppression of the TSC1/TSC2 complex and activation of Rheb GTPase, which in turn stimulates the TOR pathway [43, 83]. Simultaneously, AKT activation prevents the transcription factor forkhead box O (FOXO) from translocating into the nucleus, attenuating its transcriptional regulation of immune-related genes [23]. Amino acid-dependent TOR signaling and hormonal inputs (JH and 20E) coordinately regulate ILP signaling in a stage-dependent manner [18, 65]. CRISPR-Cas9 knockout studies demonstrate that *ILP-2* mutants exhibit diminished lipid reserves, and deletion of *ILP-4* and *ILP-5* impairs yolk formation, emphasizing the critical role of ILPs in regulating energy balance during oogenesis [18]. Species-specific ILP diversity suggests evolutionary divergence in immune–reproductive coupling. *Ae. aegypti* encodes eight ILPs, compared to five in *Anopheles* species, and three to seven in *Culex pipiens* and *Aedes albopictus* [84]. In *D. melanogaster*, insulin receptor (*INR*) mutations cause defective oocyte development and yolk deposition, ultimately resulting in infertility [85]. Similarly, in *Ae. aegypti*, INR expression patterns and phosphorylation levels correlate with ovarian maturation and steroid hormone biosynthesis [86]. Post-blood meal, IIS coordinates with the TOR pathway to regulate nutrient utilization by inhibiting glycogen synthase kinase 3 (GSK3), thereby promoting oocyte proliferation [19, 87]. Notably, the regulatory effects of IIS showed marked changes under different physiological conditions: for example, in the diapause state of *Culex pipiens*, IIS signaling is weakened with shortened daylight hours, resulting in impaired ovarian development and increased fat reserves [88].

Furthermore, IIS shapes anti-pathogen defenses through multiple downstream effectors and extensive signaling cross talk. Overexpression of phosphatase and tensin homolog (*PTEN*) in *An. stephensi* significantly inhibits *P. falciparum* development, suggesting that *PTEN* may indirectly affect immune responses by promoting autophagy and stem cell activity [89]. Similarly, AKT signaling overactivation decreases the parasite burden and infection prevalence, highlighting the context-specific immunomodulatory role of IIS [90]. IIS regulates flavivirus infections, including DENV, ZIKV, and West Nile virus (WNV), by activating the JAK-STAT pathway through the ERK pathway [24]. Low levels of human insulin-like growth factor 1 (IGF1) support midgut homeostasis and parasite resistance, whereas high levels induce oxidative stress and disrupt epithelial integrity [91]. In *Drosophila*, IIS modulates immunity via FOXO regulation: FOXO overexpression suppresses viral replication and induces ATGs such as *Atg6*, *Atg7*, *Atg8,* and AMPs [92]. However, chronic FOXO activation in aged flies suppresses peptidoglycan recognition protein SC2 (PGRP-SC2), impairing microbial recognition and causing immune dysregulation [93]. Intriguingly, IIS-TOR pathway perturbation also affects sex-biased gene expression in *Drosophila*, suggesting that IIS may contribute to physiological trade-offs, sexual conflict, and gene regulation [94].

### Autophagy-mediated immune–reproductive modulation and the role of mosGILT

Autophagy, a conserved lysosomal degradation process essential for cellular homeostasis and immune regulation across metazoans [25], plays a pivotal role in coordinating mosquito reproductive output and immune defense through nutrient-sensing pathways such as TOR/S6K (S6 kinase) [26, 95, 96]. In *Ae. aegypti*, programmed autophagy in the fat body enables timely termination of Vg synthesis and facilitates progression to the second reproductive cycle, with the E93 regulatory factor likely playing a crucial role in this process [26, 97]. In addition, the unfolded protein response (UPR) activates autophagy during vitellogenesis; RNAi-mediated silencing of UPR-related genes impairs mosquito fertility, accompanied by a marked reduction in the expression of autophagy-specific markers [27].

Autophagy also exerts a significant regulatory role in mosquito defense against infections. In *Anopheles aquasalis*, infection with *P. vivax* triggers midgut autophagic activation, which significantly reduces parasite prevalence and intensity [98]. Knockout of autophagy-related gene 8 (*AaAtg8*) in *Ae. albopictus* compromises antibacterial defenses in the midgut, highlighting the importance of autophagy in maintaining microbiome homeostasis [99]. However, the role of autophagy in arboviral infections is more nuanced than initially perceived. In *Ae. aegypti*, the salivary protein allergen-1 (AaVA-1) interacts with the autophagy inhibitor leucine-rich pentatricopeptide repeat-containing protein (LRPPRC) to competitively release Beclin-1 and activate autophagy, thereby promoting DENV and ZIKV replication [100]. These findings emphasize the dualistic function of autophagy, favoring immune protection against parasites and bacteria and potentially enhancing viral infection depending on the molecular context [100–103].

Beyond canonical regulation by nutrients and hormones, the cellular redox status also orchestrates mosquito reproductive and immune homeostasis [104, 105]. Specifically, oxidative stress—especially under disrupted thiol balance—can initiate autophagy via cytoplasmic translocation of high mobility group box 1 (HMGB1), linking redox signaling to autophagic activation [104, 105]. Within this regulatory framework, the *mosGILT* gene, originally identified in the salivary glands of *An. gambiae* and homologous to mammalian gamma-interferon-inducible lysosomal thiol reductase (*GILT*), has attracted attention due to its dual role in immunity and reproductive biology [106]. Although traditionally linked to antigen processing, *mosGILT* deletion in *An. gambiae* induces broad transcriptomic alterations, including enhanced expression of immune-related genes and impaired ovarian development, which are associated with reduced 20E production [107, 108]. These effects coincide with the upregulation of nucleocytoplasmic transport pathways and thioester-containing protein 1 (TEP1)-mediated immune activation [107, 108], suggesting that mosGILT interacts with redox-sensitive signaling networks that influence both autophagy and reproductive function. The ability of mosGILT to bind *Plasmodium* sporozoites and regulate their motility further emphasizes its multifunctional regulatory role across vector–parasite interactions [109]. Although the direct mechanistic links between mosGILT and autophagy remain to be elucidated, current evidence supports their relevance at the crossroads of immunity, oxidative signaling, and reproductive capacity (Fig. 2).

### Dual role of metal ions in mediating reproductive and immune dynamics

While intracellular signaling pathways such as TOR and IIS orchestrate reproductive–immune integration in mosquitoes, they do not operate in isolation. Trace metal ions, which serve as biochemical integrators of external environmental conditions and internal physiological states, represent an underappreciated but essential regulatory layer (Table 1). Far from mere passive nutrients, metal ions actively contribute to shaping the reproductive and immune capacities of mosquitoes through redox regulation, enzymatic activation, and transcriptional modulation [110, 111]. Recent reviews indicate that multiple metal-binding proteins and metal-dependent enzymes participate in both reproductive processes and innate immune responses, underscoring that metal homeostasis actively integrates these physiological systems rather than functioning solely as a nutritional source [28].

Iron homeostasis is pivotal for mosquito reproductive capacity. Following blood feeding, excess iron must be tightly controlled to mitigate oxidative stress [112]. In *Ae. aegypti*, the Malpighian tubule–enriched transporter *dyspepsia*, strongly induced after blood meals, facilitates systemic iron redistribution and detoxification. RNAi-mediated silencing of *dyspepsia* disrupts iron homeostasis, reduces ferritin expression, and leads to reduced fecundity, decreased hatching rates, and abnormal egg pigmentation [113]. In addition to membrane transporters that contribute to post-blood-meal iron handling and detoxification, transferrins mediate systemic iron trafficking. In *Anopheles culicifacies*, functional suppression of a transferrin homolog significantly reduces fecundity [30]. Beyond iron, the metals cadmium (Cd), zinc (Zn), and copper (Cu) also exert significant reproductive effects. At 5 ppm, Cd impaired fecundity and embryonic hatchability in both *Ae. albopictus* and *An. gambiae* [114]. In contrast, zinc levels fluctuate dynamically during *Drosophila* oocyte maturation and fertilization, highlighting its supportive role in reproduction [115]. Copper exhibits a biphasic pattern: LC50-level exposure disrupts Malpighian tubule function and ovarian development in female mosquitoes, whereas non-toxic concentrations are essential for eggshell formation and follicular maturation [116]. Together, these findings suggest that trace metal ions act not only as cofactors but also as regulators of reproductive physiology, responsive to internal metabolic states and environmental exposures.

Although trace metal ions are increasingly recognized as pivotal regulators of mosquito reproductive physiology, modulating gene expression, oogenesis, and egg viability, they also critically influence immune function through ROS/redox signaling and enzymatic cofactor activity. More broadly, metal ions influence immune competence by shaping cellular redox balance and serving as indispensable cofactors for metalloenzymes involved in host defense; for example, Zn and Cu support multiple enzyme systems linked to antimicrobial responses, whereas manganese (Mn) primarily supports antioxidant protection as a cofactor for Mn-dependent superoxide dismutase [110, 117]. As a core component of the active center of phenol oxidase (PO), Cu ions regulate the melanization biosynthetic process and modulate the activity of immune effectors to defend against pathogen infections [29, 118]. Chelation of Cu and Fe in *Anopheles albimanus* restricts *Plasmodium* parasite growth within the host [31]. Iron availability must be tightly balanced during infection. In *Ae. aegypti*, elevated host-derived serum iron enhances ROS production and reduces dengue virus infection [32]. Conversely, excessive iron availability has been linked to increased malaria risk and severity, highlighting the dual role of iron in host-parasite interactions [119]. The host proteins transferrin and serum ferritin sequester Fe, restricting its availability to pathogens and thus actively mediating the anti-*Plasmodium* defense [120]. Consistent with the concept of “nutritional immunity” [121], regulating iron availability may serve as a key interface linking metabolic homeostasis and immune defense in mosquitoes. Mn ions have emerged as key signaling molecules in antiviral immunity. Viral infection disrupts the mitochondrial membrane potential and triggers organelle acidification, resulting in the release of Mn ions as an endogenous signal that activates the cGAS-STING pathway. Circulating Mn ions taken up by immune tissues further amplify the innate responses [122]. Whether analogous Mn-dependent potentiation functions in mosquitoes remains to be determined.

## Conclusions

Mosquito reproduction and innate immunity are both resource-demanding processes that compete for limited nutritional and energetic reserves. Consequently, dynamic resource allocation across tissues must be tightly coordinated throughout the gonotrophic cycle. Endocrine signals (JH, 20E) cross talk with nutrient-sensing pathways (IIS-TOR, AMPK) and regulatory processes including autophagy and metal homeostasis to balance fecundity and immune defense. This integrated regulation relies largely on inter-organ communication: the midgut governs nutrient acquisition and immune homeostasis, the fat body acts as the central metabolic hub, and the ovary functions as the terminal reproductive sink. Blood meal-derived resources are routed through the gut–ovary and fat body–ovary axes to drive vitellogenesis and oocyte maturation while fine-tuning immune activity. Despite these advances, the specific mechanisms by which infection redirects resource allocation between reproduction and immunity through reshaping the mosquito’s metabolic network remain to be elucidated. Future studies integrating multi-omics with tissue and time-resolved functional genetics are urgently needed to define actionable core nodes and critical windows. Importantly, future research should move beyond descriptive frameworks toward intervention-oriented strategies. In particular, microbiome-based vector control approaches—such as manipulation of gut microbial communities, symbiont engineering, and microbiota-mediated immune priming—represent promising avenues to modulate host metabolic and immune states. Such an approach would enable stratified modulation of fecundity, immune competence, and vectorial capacity, thereby linking mechanistic insights into immune–reproductive trade-offs with practical strategies for transmission control. It further delineates key control points within transmission-related networks and provides a framework for target validation and intervention optimization.

## Data Availability

Data supporting the main conclusions of this study are included in the manuscript.

## Abbreviations

20E: 20-Hydroxyecdysone
3D20E: 3-Dehydro-20-hydroxyecdysone
AaAtg8: *Aedes albopictus* Autophagy-related gene 8
AaVA-1: *Aedes aegypti* Venom allergen-1
*Ae. aegypti*: *Aedes aegypti*
*Ae. albopictus*: *Aedes albopictus*
AMPK: AMP-activated protein kinase
AMPs: Antimicrobial peptides;
*An. gambiae*: *Anopheles gambiae*
*An. stephensi*: *Anopheles stephensi*
ATGs: Autophagy-related genes
BCAT: Branched-chain amino acid aminotransferase
BrZ2/BrZ4: Broad-complex zinc finger protein 2/4
βFTZ-F1: Beta isoform of Fushi tarazu factor-1
CA: Corpora allata
CHIKV: Chikungunya virus
DENV: Dengue virus
E93: Ecdysone-induced protein 93F
E75-RD: Ecdysone-induced protein 75 isoform RD
EcR: Ecdysone receptor
FOXO: Forkhead box O
GATAa: GATA transcription factor a
GATAr: GATA transcription factor r
GPCRs: G protein-coupled receptors
GSK3: Glycogen synthase kinase 3
HMGB1: High mobility group box 1;
HR3: Hormone receptor 3
IGF1: Insulin-like growth factor 1
ILPs: Insulin-like peptides
INR: Insulin receptor
IIS: Insulin/insulin-like growth factor signaling
JAK-STAT: Janus Kinase-Signal Transducer and Activator of Transcription
JHAMT: Juvenile hormone acid methyltransferase
JH: Juvenile hormone
JNK: C-Jun N-terminal kinase
Kr-h1: Krüppel homolog 1
Lp: Lipophorin
LRIM: Leucine-rich repeat immune protein
LRPPRC: Leucine-rich pentatricopeptide repeat-containing protein
Met: Methoprene-tolerant
mJHBP: Mosquito juvenile hormone-binding protein
mosGILT: Mosquito gamma-interferon-inducible lysosomal thiol reductase
miRNAs: MicroRNAs
NF-κB: Nuclear factor kappa-light-chain-enhancer of activated B cells
OEH: Ovarian ecdysteroidogenic hormone
pJNK: Phosphorylated JNK
piRNA: Piwi-interacting RNA
PAMPs: Pathogen-associated molecular patterns
PE: Pre-blood meal stage
PGRP-LC: Peptidoglycan recognition protein LC
PGRP-SC2: Peptidoglycan recognition protein SC2
PO: Phenoloxidase
PPO: Prophenol oxidase
PTEN: Phosphatase and tensin homolog
*P. gallinaceum*: *Plasmodium gallinaceum*
*P. falciparum*: *Plasmodium falciparum*
*P. vivax*: *Plasmodium vivax*
RNAi: RNA interference
ROS: Reactive oxygen species
RpL23: Ribosomal protein L23
RpL27: Ribosomal protein L27
S6K: S6 kinase
siRNAs: Small interfering RNA
STAT: Signal Transducer and Activator of Transcription
Tai: Taiman
TOR: Target of rapamycin
TEP1: Thioester-containing protein 1
UPR: Unfolded protein response
USP: Ultraspiracle
Vg: Vitellogenin
VgR: Vg receptor
WNV: West Nile virus
ZIKV: Zika virus
